# Handheld Ultra-Fast Duplex Polymerase Chain Reaction Assays and Lateral Flow Detection and Identification of *Leishmania* Parasites for Cutaneous Leishmaniases Diagnosis

**DOI:** 10.3390/pathogens12111292

**Published:** 2023-10-28

**Authors:** Insaf Bel Hadj Ali, Yusr Saadi-Ben Aoun, Zeineb Hammami, Oumayma Rhouma, Ahmed Sahbi Chakroun, Ikram Guizani

**Affiliations:** Laboratory of Molecular Epidemiology and Experimental Pathology-LR16IPT04, Institut Pasteur de Tunis, University of Tunis El Manar, Tunis 1002, Tunisia; yusrsaadi@yahoo.fr (Y.S.-B.A.); zeineb.hammami@insat.u-carthage.tn (Z.H.); oumaymaj94@gmail.com (O.R.); ahmed.chakroun@gmail.com (A.S.C.); iguizani@yahoo.com (I.G.)

**Keywords:** cutaneous leishmaniases, molecular diagnosis, point of care, *Leishmania*, molecular target, Palm PCR, duplex PCR, lateral flow immunoassay

## Abstract

Early and accurate detection of infectious diseases is a key step for surveillance, epidemiology and control, notably timely disease diagnosis, patient management and follow-up. In this study, we aimed to develop handheld ultra-fast duplex PCR assays coupled to amplicon detection by lateral flow (LF) immunoassay to deliver a rapid and simple molecular diagnostic test for concomitant detection and identification of the main *Leishmania* parasites encountered in Tunisia. We selected two DNA targets to amplify *L. major*/*L. tropica* and *L. infantum*/*L. tropica* groups of species DNAs, respectively. We optimized the experimental conditions of a duplex ultra-fast PCR. The amplification is performed using a portable Palm convection PCR machine within 18 min, and the products are detected using an LF cassette within 10 min. The test allows the identification of the infecting species according to the position and number of test lines revealed. Tested on a selection of DNAs of representative *Leishmania* strains of the three studied species (*N* = 37), the ultra-fast duplex PCR–LF showed consistent, stable and reproducible results. The analytical limit of detection of the test was 0.4 pg for *L. major*, 4 pg for *L. infantum* and 40 pg for *L. tropica*.

## 1. Introduction

Early and accurate detection of infectious diseases is a key step for surveillance, epidemiology and control but notably for timely disease diagnosis, patient management and follow-up. According to the World Health Organization Special Program for Research and Training in Tropical Diseases (WHO/TDR), an ideal diagnostic tool should be used at point of care (POC) and fulfill the ASSURED criteria: Affordability, Sensitivity, Specificity, User friendliness, Rapidity and Robustness, Equipment-free and Deliverable to end-users. During the last decade, several diagnostic tests satisfying these criteria were developed to identify major human pathogens such as human immunodeficiency virus (HIV), tuberculosis (TB) and malaria [1], as these diseases received much attention compared to neglected tropical diseases such as leishmaniases. Leishmaniases are a group of vector-born parasitic diseases with a wide range of clinical manifestations ranging from self-healing cutaneous lesions to disfiguring muco-cutaneous ulcers to visceral leishmaniasis (VL) known also as kala-azar, which is fatal if left untreated. Cutaneous leishmaniases (CL) represent a major but worldwide neglected public health problem. In the Old World (OW), more than one million CL cases are annually reported and 80% occur in the Middle East and North Africa (MENA) region. Challenges in clinical CL patient management are essentially due to diverse clinical manifestations, multiple causing agents and their co-endemicity. They are complicated by the continuous change in *Leishmania* epidemiology in Tunisia [2,3] and worldwide [4,5,6,7,8], making surveillance, epidemiology and disease control challenging. In addition, the primary drugs employed for CL treatment are toxic and their efficiency may depend on parasite species/strains [9,10,11], which emphasizes even more on the relevance of CL etiology. However, CL diagnosis is routinely performed via microscopy direct examination on Giemsa-stained smears, a time-consuming technique that needs trained personnel and cannot identify the parasites. Species identification and taxonomical differentiation can only be performed via molecular tests. Conventional polymerase chain reaction (PCR) is the molecular gold standard technique used to detect the parasites but should be complemented in a second step using other lengthy and laborious tests (Restriction Fragment length polymorphism (RFLP), sequencing…) for species identification [12,13]. The PCR ITS1 followed by RFLP developed by [12] is still used in recent studies to detect and identify *Leishmania* parasites [14]. Other PCR-based methods were also used to distinguish between *Leishmania* species. A recent study based on a real-time PCR was able to distinguish between *L. infantum* and *L. donovani* for VL and between *L. major*, *L. tropica* and *L. infantum*/*L. donovani* for CL [15]. Another study based on PCR High-Resolution Melting analysis (HRM) was also established to distinguish between *L. major* and *L. tropica* in cutaneous lesions [16]. However, all these techniques are lengthy, require sophisticated equipment and need to be performed in well-equipped laboratories. At present, there is only one commercially available CL diagnosis tool (CL Detect™ Rapid Test, InBios, Seattle, USA) meeting POC criteria for generic *Leishmania* detection. It is a lateral flow-based immunoassay that detects amastigotes antigens present in skin lesions of individuals infected with *Leishmania* parasites. However, this test was not recommended for use by some studies [17]; or it was shown that it should be complemented by additional methods because of its low sensitivity [18]. Other POC format tools based on isothermal amplification were also developed [19,20,21,22,23,24]. Nevertheless, all these tools are generic and detect *Leishmania* parasites without identifying them. Consequently, a simple, reliable and rapid DNA test that detects and identifies the species while minimizing time to result does not yet exist for CL.

Despite PCR being a laboratory-based technique, advances in technologies adapted its use for POC testing. In addition, recent pandemics and outbreaks have given us a clear reminder that there is an increasing need for portable PCR solutions for remote testing for surveillance and diseases control. Palm PCR is a battery-powered and pocket-sized convective PCR machine able to perform DNA amplification in ultra-fast speed (10–18 min). Using a ready-to-use mix for amplification and lateral flow immunoassays for *Leishmania* parasites detection makes the Palm PCR a very promising option for on-site testing [25,26,27].

Therefore, in this study we aim to deliver novel CL molecular diagnosis assays that satisfy POC criteria for timely patient management and disease control. Indeed, this study describes handheld ultra-fast duplex PCR assays coupled to amplicon detection by lateral flow (LF) chromatography on a generic cassette (PCRD). We demonstrated their potential as rapid and simple molecular diagnostic tests for the concomitant detection and identification of the main *Leishmania* parasites encountered in Tunisia and the Old World including *L. major*, *L. tropica* and *L. infantum/donovani*. Our test intends to equip areas with low resources and poor laboratories infrastructure with equitable access to high-quality patient diagnosis and management.

## 2. Materials and Methods

### 2.1. Ethical Statement

The study is approved by the Ethic Committee of Institut Pasteur de Tunis (Ref:2016/24/I/LRIPT04).

### 2.2. Parasite Strains

We used a selection of 37 well-characterized *Leishmania* DNAs belonging to *L. major* (*N* = 12), *L. infantum* (*N* = 11), *L. tropica* (*N* = 10), *L. donovani* (*N* = 1), *L. aethiopica* (*N* = 1), *L. arabica* (*N* = 1) and *L. turanica* (*N* = 1) species for test development (Table 1). Analyzed DNAs were extracted from *Leishmania* strains obtained from reference centers in Monpellier, clinical isolates from health centers in Tunisia and strains isolated from reservoirs in the frame of field study in Tunisia [28]. Species assignment was undertaken by isoenzyme and/or PCR and RFLP typing [12].

### 2.3. Assays Design

**Figure 1:**
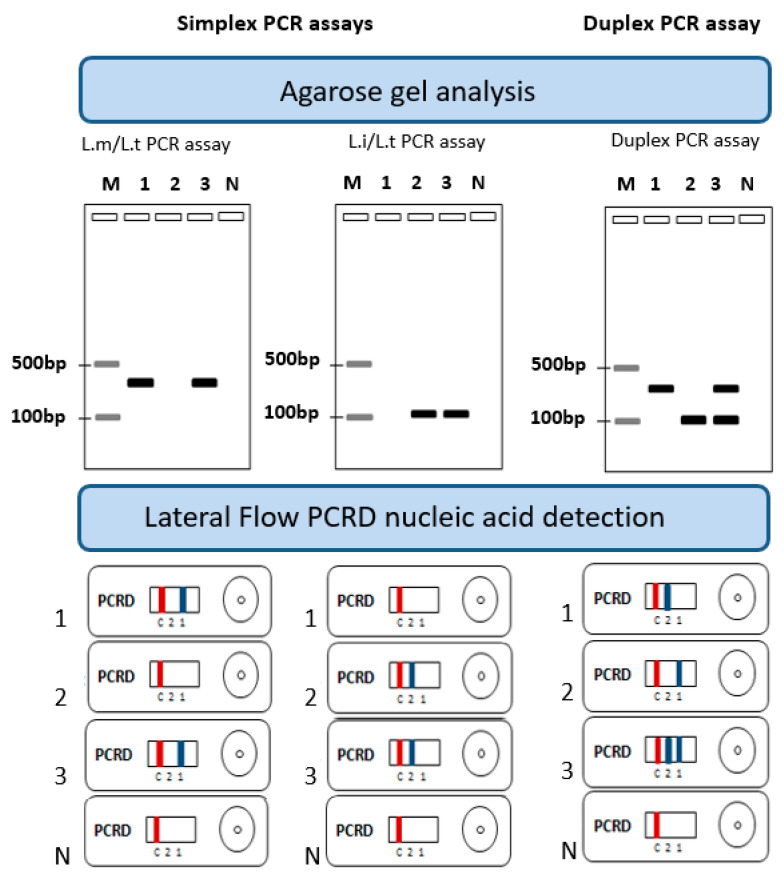
Principles underlying simplex and duplex ultra-fast PCR and lateral flow PCRD assays for the simultaneous detection and identification of *Leishmania* parasites and expected results. 1: *L. major* (*L.m*), 2: *L. infantum* (*L.i*), 3: *L. tropica* (*L.t*), N: no template control, M: molecular weight, PCRD test line 1 detects *L. major*/*L. tropica* (*L.m*/*L.t*) using DIG/Biotin-labeled primers, PCRD test line 2 detects *L. infantum*/*L. tropica* (*L.i*/*L.t*) using FAM/Biotin-labeled primers, and C: control line.

We aimed to design assays based on ultra-fast PCRs coupled to PCRD lateral flow for the concomitant detection and identification of the 3 studied *Leishmania* species (*L. major*, *L. tropica* and *L. infantum*). As the PCRD (and most of the commercially available lateral flow test) has only two test lines, we sought to identify two groups of DNA targets. The first group corresponded to DNA targets showing higher levels of similarity in their DNA sequences between *L. major* and *L. tropica* as compared to *L. infantum* to identify an *L. major*/*L. tropica*-specific target. The second group showed higher levels of similarity in their DNA sequences between *L. infantum* and *L. tropica* as compared to *L. major* to identify an *L. infantum*/*L. tropica*-specific target. The two specific targets were first used to set up groups of species-specific simplex assays that will be than combined for the set up of an ultra-fast/LF duplex assay. Lateral flow detection is a sandwich immunochromatografic-based assay that relies on the appropriately labeled primers (FAM/Biotin and DIG/Biotin) used for the PCR assays. The labels we used were recommended by the PCRD manufacturer so that the labeled amplicons were captured by the antibodies immobilized at the test lines (anti-Biotin) to form a colored complex and, therefore, a visible line. Principles underlying the ultra-fast PCR and LF assays for the simultaneous detection and identification of *Leishmania* parasites and the expected results are shown in Figure 1.

### 2.4. Target Selection and Primers Design

DNA targets were identified based on bibliography search, were selected essentially from specific genes published by [29], listed among the species-specific ones [29] or genes used in other published molecular methods and were described as showing inter-species sequence polymorphisms [30] (Table 2). Sequences of these targets were available in our local database in addition to those retrieved from TritrypDB public database (Release 59, 30 August 2022). We used Geneious 3.6.2 computer program (Biomatters, Inc, Boston, MA, USA) for sequence alignment and SNPs identification.

Primers were designed manually to specifically amplify DNA targets in *L. major*/*L. tropica* or *L. infantum*/*L. tropica* groups of species. The design took in consideration sequence polymorphisms across multiple sequence alignments of strains and species. Priming sites were selected to be conserved within a group of species (e.g., *L. major*/*L. tropica*) and present polymorphisms in the DNA sequences of the remaining studied species (e.g., *L. infantum*). Moreover, primers design was undertaken considering other criteria including expected amplicon size (should be different for the two DNA targets involved in the duplex PCR), melting temperature (should be the same for the two DNA targets involved in the duplex PCR) and the absence of secondary structures and internal hybridization for optimal PCR efficiency.

### 2.5. Ultra-Fast Simplex PCR Assays for Target Screening

To screen for *Leishmania* group of species simplex specific amplification assays, we used non-labeled primers (RAN Biolinks, Tunis, Tunisia) (Table 3) and agarose gels visualization; the retained primers were then labeled to be able to detect the amplicons via lateral flow assay.

The 20 µL PCR mixture contained a ready-to-use 1× PalmTaq Express Master Mix (Ahram Biosystems, Inc., Seoul, Republic of Korea) that includes 0.8 U hot start PalmTaq high-speed DNA polymerase, 2.5 mM MgCl_2_ and 0.2 mM dNTPs, to which we added 0.5 µM each primer and 2 µL of 20 ng/µL parasite’s DNA. PCR was run in a battery-operated Palm PCR G3 ultra-fast mobile PCR system (Ahram Biosystems, Inc., Seoul, Republic of Korea). In the Palm PCR system, each run was controlled by a protocol, as defined by a set of control parameters. A PCR protocol is defined by four control parameters including PCR speed or Turbo (T1, T2 or T3), annealing temperature (52–60 °C), cycle’s number (max 100) and a preheat step. After selecting a protocol on the Palm PCR device, the PCR time was automatically set by the operating software linked to the speed and cycles, respectively. The duration of the PCR was 10-18 min depending on the Turbo (T) and the cycle’s number selected. We used Turbo 3 (T3) and 45 cycles to run our simplex PCRs. Annealing temperature was set depending on the primer pairs used. The reaction time was 13 min 30 s. Then, PCR products were analyzed using 2% agarose gel electrophoresis (1 h, 80 V). For each primer pair, reactivity was tested on a selection of 3 strains’ DNA per each species, and the selection was based on their ability to react according to taxonomic specificity.

### 2.6. Ultra-Fast Duplex PCR Assays Screening and Optimization

To set up duplex assays, most relevant simplex assays were engaged in ultra-fast duplex PCR where 2 primer pairs were added in the same reaction mix. Ultra-fast duplex PCR highly depends on the target sequence, amplicon size and primers properties. Therefore, different primers combinations (Table 4), equimolar concentrations (0.5 µM, 0.4 µM, 0.3 µM, 0.25 µM and 0.2 µM) and ratios (0.35:0.15 and 0.3:0.2) were tested in order to select primers combination/concentration having the best analytical sensitivity and specificity. The reactions were set up at room temperature and run using the battery-powered Palm portable PCR machine (Ahram Biosystems Inc., Seoul, Republic of Korea). We used Turbo 1 (T1), 45 cycles and an annealing temperature of 60 °C to run our PCRs. PCRs run time using T1 speed and 45 cycles parameters was 18 min. Visualization of the PCR products was undertaken using agarose gel electrophoresis (1 h, 80 V).

### 2.7. Ultra-Fast Duplex PCR and LF Reading

The 20 µL PCR mixture contained a ready-to-use 1× PalmTaq Express Master Mix (Ahram Biosystems, Inc., Seoul, Republic of Korea), to which we added 0.3 µM of primers amplifying mt30 marker, 0.2 µM of primers amplifying it20 marker and 2 µL of 20 ng/µL parasite’s DNA. The ultra-fast duplex PCR was run in the Palm PCR device employing the previously described control parameters (T1, 45 cycles, Ta = 60 °C). Then, 6 µL of the PCR products were diluted in 84 µL dilution buffer (Abingdon Health, York, UK), 75 µL of which were transferred in the sample pad of the PCRD cassette as recommended by the PCRD manufacturer. Amplicons were then captured on a two-test lines neutravidin-coated carbon nanoparticle-based LF chromatography system (PCRD, Abingdon Health, York, UK). The result was read with the naked eye after 10 min flow migration, and a picture was immediately taken for our records.

### 2.8. Analytical Specificity and Sensitivity

The retained ultra-fast PCR protocol using the retained primer pairs was validated for its taxonomic specificity on panels of representative well-described *Leishmania* strains belonging to different species from diverse geographical origins and hosts (Table 1). Analytical sensitivity was also tested on 1/10 serial dilutions starting from 20 ng/µL to 2 × 10^−5^ ng/µL of input DNA of *Leishmania* parasites belonging to the 3 species: *L. major*, *L. tropica* and *L. infantum.*

To mimic a real situation of the detection of parasites’ DNA from CL samples and test the impact of human DNA on our ultra-fast PCR assays, 50 µL of human blood was spiked with 4 × 10^8^ of *L. major*-cultured promastigotes. Then, total DNA (human and parasite’s DNAs) was extracted using QIAmp DNA mini kit (Qiagen, Venlo The Netherlands) as recommended by the manufacturer. The extracted DNA was 10-fold serially diluted and used to investigate the analytical sensitivity of our developed ultra-fast duplex PCR/LF test.

## 3. Results

### 3.1. Target Selection and Primers Design

**Figure 2:**
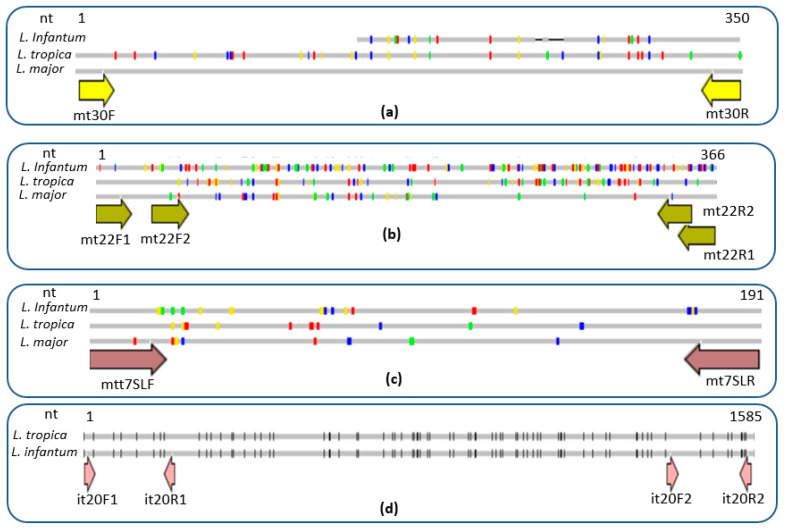
Schematic representation of sequences alignment of the selected DNA targets of the studied *Leishmania* species representatives and primers position (**a**) mt30 target: the first part of the sequence is missing in *L. infantum*; (**b**) mt22 target; (**c**) mt7SL target; (**d**) it20 target: the sequence is absent in *L. major*. Black and colored dots represent the mismatches between sequences, gray lines are conserved nucleotides and nt: nucleotide.

Based on multiple alignments of *Leishmania* species DNA sequences, we identified in total four promising DNA targets with relevance to develop our expected specific ultra-fast PCR assays. They correspond to coding and intergenic regions with a high level of conservation (>90%) between *L. major* and *L. tropica* DNA sequences (mt30, mt7SL and mt22) and others with a high level of conservation (>90%) between *L. tropica* and *L. infantum* DNA sequences (it20). Our local database from an ongoing project contained sequence information covering different strains of the species of interest. Within these targets, we searched for sequence polymorphisms to design primer pairs specific to the *L. major*/*L. tropica* group of species and others specific to the *L. infantum*/*L. tropica* group of species. We designed in total six primer pairs, which include six primer pairs targeting the *L. major* and *L. tropica* group of species (mt30FR (Figure 2a), mt22F1R1 (Figure 2b), mt22F2R2 (Figure 2b), mt22F1R2 (Figure 2b), mt22F2R1 (Figure 2b) and mt7SLFR (Figure 2c)) and two targeting the *L. infantum* and *L. tropica* group of species (it20F1R1 (Figure 2d) and it20F2R2 (Figure 2d)) (Table 3).

### 3.2. Specific Simplex Ultra-Fast PCRs Screening Assays

For cost effectiveness, simplex PCR assays were first set up using non-labeled primers (RAN Biolinks, Tunis, Tunisia) in order to test the taxonomic specificity of the designed primers. For each primer pair, taxonomic specificity was tested on a selection of three strains for each species.

**Figure 3:**
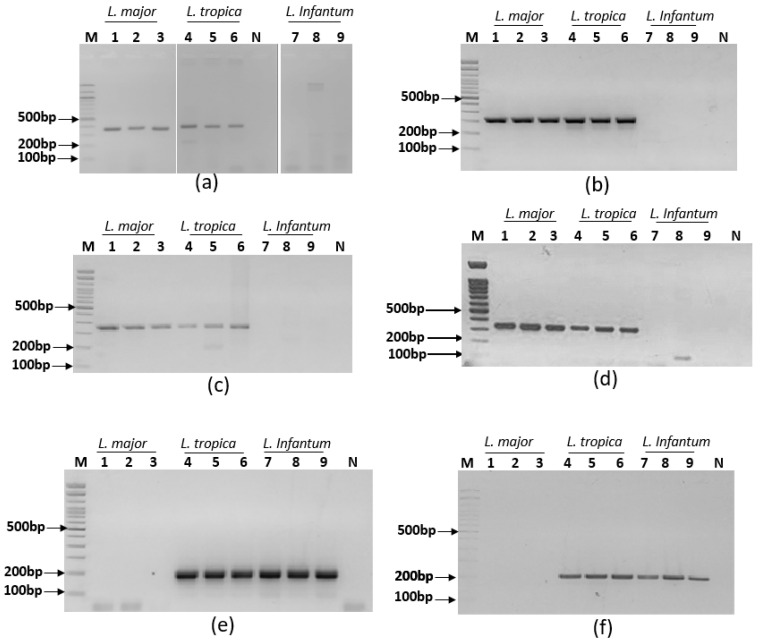
Amplification results of the selected primer pairs used for the ultra-fast simplex PCR assays set in this study visualized on a 2% agarose gel electrophoresis. (**a**) mt22F1/R1, (**b**) mt22F2/R2, (**c**) mt30F/R, (**d**) mt22F2R1, (**e**) it20F2/R2, (**f**) it20F1/R1, *L. major* (1: P-strain, 2: IL53, 3: R115), *L. tropica* (4: DBKM, 5: A Sinai III, 6: Adhanis), *L. infantum* (7: LV08, 8: LV49, 9: LV10), M: 100 bp molecular weight, N: no template control.

Simplex PCR assays were set up to target the *L. major* and *L. tropica* group of species and the *L. infantum* and *L. tropica* group of species. They were run using PalmTaq Express Master Mix (Ahram Biosystems, Inc., Seoul, Republic of Korea) and non-labeled primers in the Palm PCR device. Amplicons were visualized on agarose gels. Results showed that primer pair mt7SLFR reacted with the three tested *Leishmania* species and primer pair mt22F1R2 showed non-specific amplifications. So, these primer pairs were rejected. Six primer pairs gave specific profiles as expected. Indeed, primer pairs mt22F1R1 (Figure 3a), mt22F2R2 (Figure 3b), mt22F2R1 (Figure 3c) and mt30F/R (Figure 3d) reacted with *L. major* and *L. tropica* DNAs without reacting with *L. infantum* DNA; primer pairs it20F2/R2 (Figure 3e) and it20F1/R1 (Figure 3f) reacted with *L. infantum* and *L. tropica* DNAs without reacting with *L. major* DNA (Figure 3). These primers were therefore selected for the ultra-fast duplex PCR assays development.

### 3.3. Ultra-Fast Duplex PCR and LF Detection Assays Set Up

To set up the ultra-fast duplex PCR, we tested eight different combinations of PCRs primers including mt22F1R1/it20F1R1 (A), mt22F2R2/it20F2/R2 (B), mt22F1R1/it20F2R2 (C), mt22F2R2/it20F1R1 (D), mt22F2R1/it20F1R1 (E), mt22F2/R1/it20F2R2 (F), mt30FR/it20F1R1 (G) and mt30FR/it20F2R2 (H) (Table 4).

**Figure 4:**
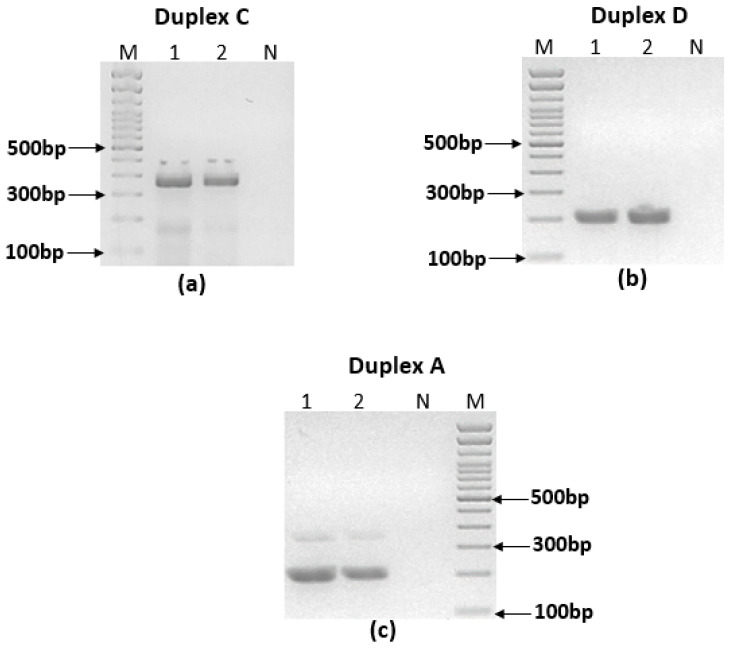
Different scenarios of ultra-fast duplex PCR with *L. tropica* species DNA using different combinations of primers. (**a**) Duplex PCR C showing non-specific amplification. (**b**) Duplex PCR D showing one band with *L. tropica*. (**c**) Duplex PCR A showing significant unbalanced amplification. 1: Bumm30, 2: DBKM, M: 100 bp molecular weight, N: no template control.

Most of the combinations in the duplex PCRs did not give the expected results especially with *L. tropica* species. Some of the combinations (C, F and H) gave non-specific amplifications (Figure 4a). In other cases, (B, D and E), we observed only one band for *L. tropica* where two bands were expected (Figure 4b). In one case (A), we observed a significant imbalance in band intensities (Figure 4c).

**Figure 5:**
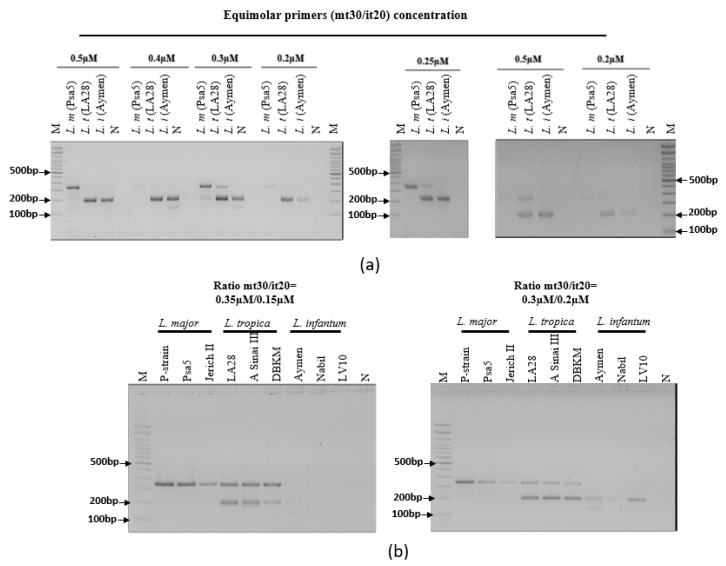
Agarose gel profiles of the mt30FR/it20F1R1 ultra-fast duplex PCR using different primer concentrations. (**a**) Using equimolar concentrations. (**b**) Using 2 different ratios. N: No template control; M: 100 bp molecular weight.

The best results obtained in terms of species identification and differentiation were observed with the ultra-fast duplex PCR combining mt30FR and it20F1R1 (G) primer pairs. The selected ultra-fast duplex PCR was optimized by varying primers concentrations (Figure 5a) and ratios (Figure 5b) to balance band intensities in agarose gel (Figure 5). The retained protocol is as follows: primers final concentrations mt30FR: 0.3 µM, it20F1R1: 0.2 µM (Figure 5b), T1 turbo, Ta = 60 °C, 45 cycles. Then, the selected primers were differently labeled (mt30F-Dig/R-Biotin and it20F1-Fam/R1-Biotin) (RAN Biolinks, Tunis, Tunisia) to allow detection by LF chromatography on the generic two-test line PCRD cassette used in this study (Abingdon Health, York, UK). The amplicons were first visualized on agarose gels and then on PCRD lateral flow.

**Figure 6:**
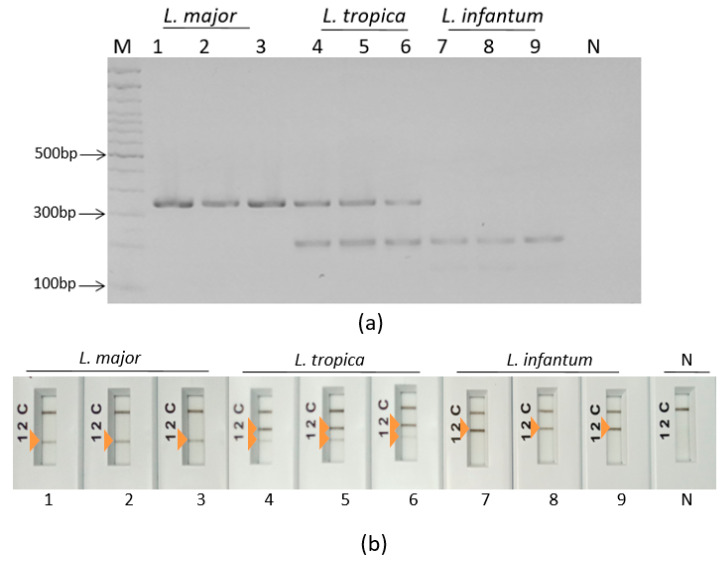
Ultra-fast duplex PCR mt30FR/it20F1R1: test of analytical specificity. (**a**) The 2% agarose gel electrophoresis. (**b**) PCRD detection. M: 100 bp molecular weight, N: no template control. 1: R44, 2: R99, 3: R155, 4: Bag17, 5: Bag9, 6: DBKM, 7: LV49, 8: D13, 9: D16, arrow: positive test.

The selected protocol was tested on a selection of 33 well-characterized *Leishmania* DNAs belonging to *L. major* (*N* = 12), *L. infantum* (*N* = 11) and *L. tropica* (*N* = 10). They showed stable and reproducible results within each species when amplicons were visualized on agarose gel (Figure 6a) as well as on PCRD lateral flow (Figure 6b).

**Figure 7:**
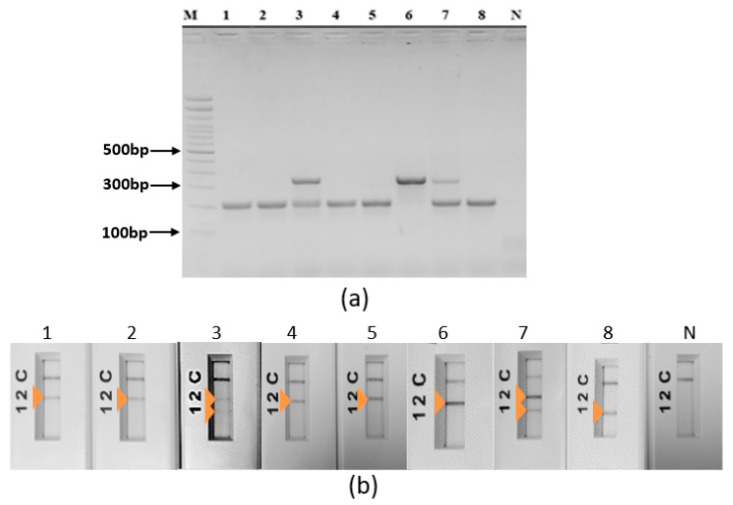
Amplification profile of other *Leishmania* species tested with the ultra-fast duplex PCR mt30FR/it20F1R1. (**a**) The 2% agarose gel electrophoresis. (**b**) PCRD detection. 1: New World *L. infantum* (PP75), 2: L. donovani (L1005), 3: L. aethiopica (L100), 4: L. arabica (J238), 5: L. turanica (95A), 6: *L. major* (R44), 7: *L. tropica* (Bag17), 8: *L. infantum* (LV49), M: 100 bp molecular weight, N: no template control, arrow: positive test.

Other *Leishmania* species were also tested including *L. donovani* (*N* = 1), L. aethiopica (*N* = 1), *L. arabica* (*N* = 1) and *L. turanica* (*N* = 1). Results showed that L. aethiopica has the same amplification profile as *L. tropica* while *L. donovani*, *L. arabica* and *L. turanica* share the same profile as *L. infantum* (Figure 7). Results shown via agarose gels (Figure 7a) are congruent with those obtained with lateral flow PCRD (Figure 7b).

**Figure 8:**
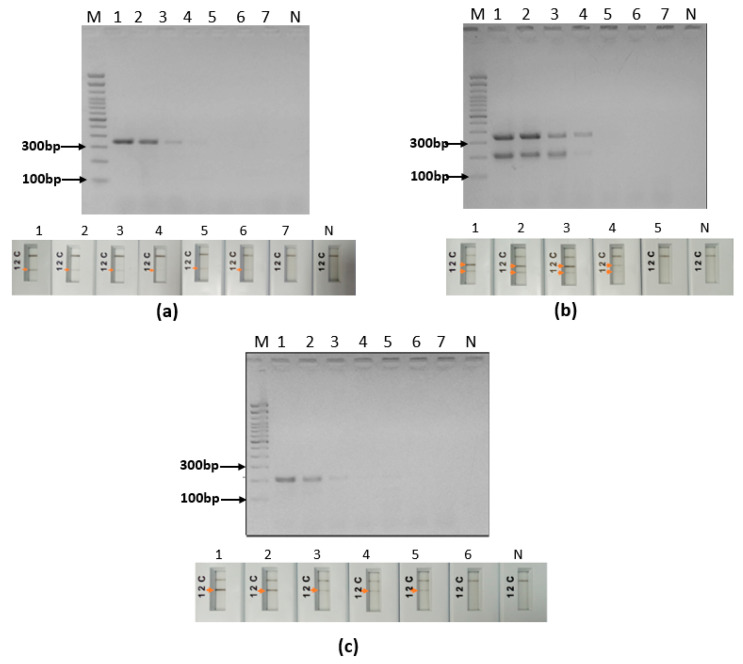
Analytical sensitivity of the ultra-fast duplex PCR mt30FR/it20F1R1 using a 10-fold serial dilution of DNA of: (**a**) *L. major*, (**b**) *L. infantum* and (**c**) *L. tropica*. M: 100 bp molecular weight, 1: 40 ng, 2: 4 ng, 3: 0.4 ng, 4: 4 × 10^−2^ ng, 5: 4 × 10^−3^ ng, 6: 4 × 10^−4^ ng, 7: 4 × 10^−5^ ng, N: no template control, arrow: positive test.

The analytical sensitivity was tested on the three species *L. major* (Figure 8a), *L. tropica* (Figure 8b) and *L. infantum* (Figure 8c) and was 0.4 pg for *L. major*, 4 pg for *L. infantum* and 40 pg for *L. tropica* (Figure 8). It corresponds to 5 parasites, 50 parasites and 500 parasites for *L. infantum*, *L. major* and *L. tropica*, respectively, if we assume an average diploid genome mass of 80 fg as stated by [31]. We notice that the test is less sensitive with *L. tropica*, as two targets are amplified in the DNA sequences of this species.

### 3.4. Impact of Human DNA on the Ultra-Fast Duplex PCR and LF Assays

**Figure 9:**
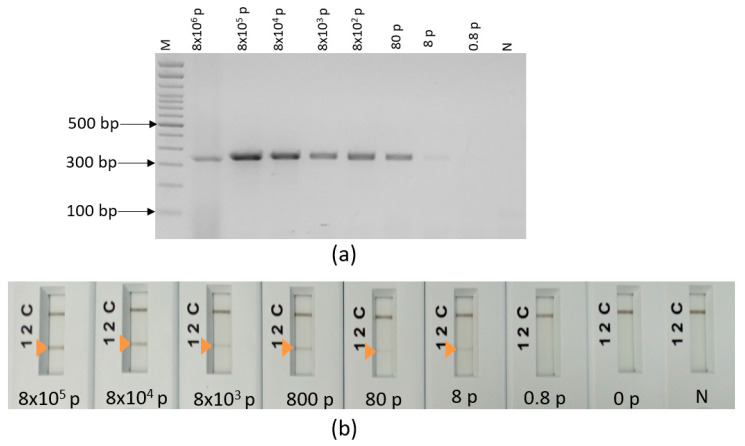
Limit of detection of the ultra-fast duplex PCR mt30F/R-it20F1R1 using a serial dilution of DNA extracted from a mixture of 4 × 10^8^ parasites and 50 µL of human blood, 2 µL of the total extracted DNA is added in the reaction. (**a**) The 2% agarose gel detection. (**b**) Lateral flow detection (PCRD), MW: molecular weight, p: parasites, N: no template control, arrow: positive test.

In order to test the impact of human DNA on our ultra-fast PCR assays and mimic a CL sample, cultured *L. major* promastigotes were spiked with human blood. Total DNA (human and parasite’s DNAs) was extracted from this reconstitution, serially diluted and used to test the analytical sensitivity of our developed ultra-fast duplex PCR/LF assay. It showed a detection limit of eight parasites in agarose gel (Figure 9a) as well as in PCRD lateral flow (Figure 9b), which is comparable to the limit of detection observed when we tested *L. major* DNA alone (five parasites). No cross-reactivity was observed when we tested our assay with human DNA.

## 4. Discussion

In this study, we aimed at developing a simple, rapid, sensitive and specific DNA-based test to detect and identify the most frequent *Leishmania* parasites causing cutaneous leishmaniasis (CL) in the OW: *L. major*, *L. tropica* and *L. infantum*. Previous studies used conventional PCR complemented by additional steps like RFLP [12] or sequencing [13] or other single-step PCR-based methods, like PCR-HRM [16] and real-time PCR [15], to identify *Leishmania species*. However, these used technologies that are not adapted for POC testing. Current advances in enzymes technology and in equipment miniaturization have made PCR rapid to perform and feasible at the POC to guide decisions on treatment and clinical management of infectious diseases [26]. Accurate detection and identification of *Leishmania* parasites in endemic areas that lack appropriate resources is a global public health problem. In Tunisia and many other countries of the OW, direct examination using microscopy is the most commonly used technique. It is specific but lacks sensitivity in addition to the fact that it does not allow for species identification. Molecular methods are needed to fill this gap, but their use is limited to few reference laboratories due to the cost of the required equipment to perform such experiments. Therefore, there is an urgent need for simple, rapid and accurate tests with high sensitivity and specificity that can be used at the point of care, without requiring special expertise nor sophisticated equipment, given the conditions prevailing in many disease-endemic areas.

Our study focused on the development of an alternative to the conventional PCR, based on duplex convective PCR using the Palm PCR device. The reaction operates with battery power at room temperature and uses a very simple system with ready-to-use mixes. It also has the advantage of being fast since the reaction takes place in 18 min (equivalent to 45 cycles of amplification); hence, it is considered an ultra-fast method. The amplicons visualization is performed using PCRD lateral flow immunoassay in 10 min. To our knowledge, this is the first study that associates convective PCR technology with lateral flow detection for *Leishmania* parasites detection and identification. The unique study describing an ultra-fast PCR for CL diagnosis used an E-gel reader electrophoresis system for the detection of *Leishmania viannia* and some mimickers such as fungal or mycobacterial infections [32]. Moreover, it is the fastest PCR-based technology method so far that could be used for the concomitant detection and identification of *Leishmania* parasites. Indeed, a conventional PCR reaction and other PCR-based techniques used for the same purpose require on average between 1.5 and 2 h for parasites detection and additional steps and time for species identification. Moreover, in our case, DNA target amplification and PCRD visualization take less than 30 min for *Leishmania* parasites detection and species identification. In the same context, a study aiming at delivering a CL diagnostic test in POC format developed a method based on recombinase polymerase isothermal amplification coupled to lateral flow detection. The test took 50 min including 40 min for the amplification and 10 min for lateral flow detection of amplicons. The described test is able to detect *Leishmania* parasites, but does not identify the species. Thus, our tool offers a time-saving edge even compared to other POC format methods.

In addition, LF testing accessibility and feasibility have been demonstrated especially during the COVID-19 pandemic. It showed to be an easy-to-use, affordable and accurate system that should be used for the next generation’s tests [33]. It offers a simple method for the decentralized diagnosis of infectious diseases and control strategies [34,35,36]. Nevertheless, coupling ultra-fast duplex PCR to LF is challenging, and the most arduous step in the development process is primer selection that avoid the formation of the non-specific band in the LF. The production of artifacts is mainly due to inter- and intra-molecular interactions of primers [37]. It is very important to carefully design and check primer properties in silico in order to maximize the probability of duplex PCR experiment success [38].

Duplex PCR success depends mainly on the target sequence, amplicon size and primers properties [37]. The selected duplex combining the primer pairs mt30F/R and it20F1/R1 allowed to have three different amplification profiles and thus to distinguish the three species studied. The difference is based on the number of amplicons and their specificities. Indeed, by using the pair of primers mt30F/R and it20F1/R1, we obtained, on agarose gel, specific profiles of species including a band of 350 bp for *L. major*, two bands of 350 bp and 209 bp for *L. tropica* and one band of 209 bp for *L. infantum*. In addition, detection of amplicons by the PCRD LF assay yielded results in agreement with those obtained on agarose gel. The LF test allows the identification of the infecting species according to the number and position of test lines revealed: *L. infantum* (line one), *L. major* (line two) and *L. tropica* (lines one and two), which makes the results read out very simple and easy to interpret. As the *L. infantum* and *L. donovani* group of species and *L. tropica* and *L. aethiopica* group of species have a high level of sequence conservation [39,40], we had PCR tests reacting similarly with the two pairs of species. This should be useful if their application would be extended to other MENA and African regions where *L. aethiopica* and *L. donovani* are predominant [41]. However, additional tests should be developed to distinguish between *L. donovani* and *L. infantum* and between *L. tropica* and *L. aethiopica* species especially in cases where each two species are co-endemic. The application of our test could be also extended to detect *Leishmania* parasites for VL or in molecular epidemiology to detect the parasites in vectors and reservoirs. Further investigations are necessary to provide evidence for this potential.

One of our primer selection criteria was that primers specificities were shared by two species like *L. major* and *L. tropica*, or by *L. tropica* and *L. infantum*. We took advantages of sequences similarity between pairs of *Leishmania* species in order to identify our DNA targets and design the group of species-specific primers. Therefore, by duplexing the two types of assays, we were able to detect and identify the three *Leishmania* species encountered in Tunisia (and in Africa and Middle East) in one single reaction. On the other hand, some combinations of primers in a duplex PCR reaction did not give the expected profile, especially for *L. tropica* where two amplicons were expected. In the majority of combinations, we observed the appearance of a single amplification band instead of two. This result could be explained by the fact that, generally, there is a certain competitiveness between primers as the two of them are competing for the same pool of reagents. There is also a preferential amplification of certain specific targets due to their GC content leading to preferential denaturation [38]; or a differential accessibility of targets within genomes due to secondary structures [38]. Therefore, a single intense band was obtained following a duplex amplification by ultra-fast convective PCR. This results in unbalanced amplifications leading to a single band or different intensities of the obtained bands [38,42]. Another hypothesis is that there is a deficiency in the spontaneous circulation of molecules using the convection principle on which Palm PCR is based. These molecules will, therefore, not be able to reach the appropriate temperature zone for the hybridization of the primer to the target. This hypothesis is supported by the fact that some of the primer combinations that performed poorly by convective PCR performed very well by conventional PCR.

Our data suggest that the ultra-fast PCR method is sensitive and that the sensitivity varies according to the species. The limit of detection of our assay is 0.4 pg for *L. major*, 4 pg for *L. infantum* and 40 pg for *L. tropica*; the equivalent of 5, 50 and 500 parasites, respectively. The test showed to be less sensitive with *L. tropica.* For this species, we had two targets competing with the same reagent mixture that reached their depletion more quickly compared to *L. major* and *L. infantum,* where a unique target was amplified. When tested on DNA extracted from human blood spiked with cultured *Leishmania* parasites, our assay detected eight parasites. We noticed that human DNA did not affect the sensitivity of our test as we had a limit of detection of five parasites when we tested our assay with *L. major* DNA. Furthermore, no cross-reactivity was observed with human DNA. Other comparable results in terms of analytical sensitivity have been obtained by other studies using different sophisticated methods. For example, a study describing a probe-based allele-specific real-time PCR for *Leishmania* species identification was able to detect 12 parasites per reaction [15]. Another study describing a multiplex PCR targeting *Leishmania* sp. kDNA and a conserved region of the mammalian gapdh gene detected 0.1 ng of *Leishmania* DNA diluted in 100 ng of mammalian DNA [43]. Nevertheless, other studies based on isothermal amplifications showed a higher analytical sensitivity by detecting as low as 0.1 parasites per reaction [44]. It is known that the sensitivity of a multiplex PCR assay is reduced with increased numbers of target genes in the reaction [45]. In a recent study describing a Palm PCR assay coupled to agarose gel electrophoresis for *Leishmania* spp., detection in cutaneous ulcers achieved a specificity and a sensitivity of 90% and 91.7%, respectively, when tested in lab conditions [32]. In the field, the same assay showed a sensitivity of 100% and a specificity of 25% [32]. The false positivity rate noted was assigned to contamination during DNA extraction in the field [32]. Our assay is combining the accuracy of a PCR test and the rapidity of an isothermal method. In addition to the time-saving Palm PCR system is a portable device that reduces the cost required for diagnostics. The average price of a conventional PCR or qPCR device is typically between USD 5000 and USD 15,000 with additional USD 0.6–2.5 for reagents per reaction, while that of Palm PCR is around USD 3000–3500 and reagents cost USD 0.6 per reaction [32]. Energy saving is also a strong point of Palm PCR. A conventional thermocycler consumes on average up to 700W of energy, whereas the Palm PCR device consumes about 5W on mains power with the possibility of operating on rechargeable battery.

The CL diagnostic test based on ultra-fast duplex PCR through the Palm PCR system and lateral flow detection by PCRD appears prone for operation in low-resource areas. This potential tool complies with the WHO “ASSURED” criteria to control and manage these infectious skin diseases beyond the framework of specialized laboratories. However, test evaluation and performance description are yet to be performed by validating our method using an adequate panel of cutaneous samples including from CL lesions. This will define the usefulness of the method and define its accuracy.

## 5. Conclusions

Through this study, we bring a proof-of-concept demonstration that an ultra-fast duplex PCR method coupled to lateral flow chromatography read out would be a valuable test that provides fast on-site sample DNA analysis with a small sample consumption, short analysis time and high sensitivity. It will potentially enable CL diagnosis at community health centers reducing the need of referring patients to a regional hospital for disease confirmation. Ultra-fast duplex PCR assays using a handheld PCR device coupled to LF read out hold promises as a valuable POC test for CL diagnosis and species-adapted therapy guidance.

## Figures and Tables

**Table 1:** Strain’s DNAs used for the tool development process.

WHO Code	Lab Code	Species	Zymodem	Clinical Manifestation
MMER/TN/87/Ron114	R114	*L. major*	MON-25	NA
MPSA/TN/87/Ron99	R99	*L. major*	MON-25	NA
MPSA/TN/87/Ron44	R44	*L. major*	MON-25	NA
MPSA/TN/87/Ron155	R155	*L. major*	MON-25	NA
MPSA/TN/87/Ron 102	R102	*L. major*	MON-25	NA
MRHO/SU/59/P-Strain	P-strain	*L. major*	MON-4	NA
MHOM/IL/83/IL24	IL24	*L. major*	MON-66	CL
MHOM/IL/83/IL53	IL53	*L. major*	MON-67	CL
MHOM/IL/67/Jericho II	Jerichll	*L. major*	MON-26	CL
MPSA/TN/89/Psa1	Psa1	*L. major*	NT	NA
MPSA/TN/89/Psa5	Psa5	*L. major*	NT	NA
MHOM/TN/11/EMPA12	EMPA12	*L. major*	NT	CL
MHOM/TN/80/IPT1	IPT1	*L. infantum*	MON-1	VL
MHOM/TN/88/Aymen	Aymen	*L. infantum*	MON-1	VL
MHOM/TN/88/Nabil	Nabil	*L. infantum*	MON-1	VL
MHOM/TN/92/LV08	LV08	*L. infantum*	NT	VL
MHOM/TN/92/LV10	LV10	*L. infantum*	MON-80	VL
MHOM/TN/94/LV49	LV49	*L. infantum*	MON-24	VL
MHOM/TN/94/LV50	LV50	*L. infantum*	MON-1	VL
MHOM/TN/97/Drep 13	D13	*L. infantum*	MON-24	CL
MHOM/TN/98/Drep16	D16	*L. infantum*	MON-24	CL
MHOM/TN87/KA412	KA412	*L. infantum*	MON-1	VL
MHOM/BR/74/PP75	PP75	*L. infantum*	MON-1	VL
MHOM/IQ/76/BAG17	Bag17	*L. tropica*	LON-24	CL
MRAT/IQ/73/Adhanis I	Adhanis	*L. tropica*	MON-5	NA
MCAN/IN/71/DBKM	DBKM	*L. tropica*	MON-62	NA
MHOM/IL/00/Gabaï159	Gabai 159	*L. tropica*	LON-9	CL
MHOM/GR/00/LA28	LA28	*L. tropica*	LON-16	CL
MHOM/IQ/73/A Sinaï III	A Sinai III	*L. tropica*	LON-11	CL
MHOM/IQ/76/BAG9	Bag 9	*L. tropica*	MON-53	CL
MHOM/SU/74/SAF K27	K27	*L. tropica*	MON-60	CL
MHOM/IQ//73/Bumm30	Bumm30	*L. tropica*	LON-17	VL
MHOM/IL/78/Rachnan	Rachnan	*L. tropica*	MON-60	CL
MHOM/ET/72/GEBRE1	L1005	*L. donovani*	MON-82	VL
MPSA/SA/84/Jisha 238	J238	*L. arabica*	LON-64	NA
MHOM/ET/72/L100	L100	*L. aethiopica*	MON-14	CL
MRHO/SU/74/95-A	95A	*L. turanica*	MON-64	NA

NA: Not applicable, NT: not typed, CL: cutaneous leishmaniasis, and VL: visceral leishmaniasis.

**Table 2:** Identified targets for the set up of the specific PCR assays.

Target	Gene			Protein
	*L. major*	*L. infantum*	*L. tropica*	
mt30	Intergenic region between LmjF30.0190 and LmjF30.0200	Intergenic region between LINF_300006850 and LINF_300006900	Intergenic region between LTRL590_300007200 and LTRL590_300007300	None
mt22	Non-coding sequence	LinJ.22.0300	LTRL590_220009300	Hypothetical protein in *L. infantum* and *L. tropica* Non-coding sequence in *L. major*
it20	Absent	LinJ.20.0040	LTRL590_200005300	Phosphate-Repressible Phosphate Permease-like protein Absent in *L. major*
mt7SL	LmjF.05.SRP.RNA	LINJ_05_snRNA1	7SL gene Partial sequence	7SL RNA

**Table 3:** Designed primer pairs and their specificity.

Primer Pairs	Sequences (5’–3’)	Size (bp)	Expected Specificity		
			*L. major*	*L. tropica*	*L. infantum*
mt22F1	ACCGAACCCAACGCTGAAG	366	+	+	-
mt22R1	AGTGCATGAGGCGTGTATGG				
mt22F2	CACTCATGCGTGTCCATTCT	319	+	+	-
mt22R2	GTATGGGAAGGTGGGGGT				
mt22F1	ACCGAACCCAACGCTGAAG	352	+	+	-
mt22R2	GTATGGGAAGGTGGGGGT				
mt22F2	CACTCATGCGTGTCCATTCT	333	+	+	-
mt22R1	AGTGCATGAGGCGTGTATGG				
mt30F	GGTGCAATGTGCGCATG	350	+	+	-
mt30R	GCTTGGCGCTCTCGAAAAG				
mt7sLF	TTGGTGGTGGTGGGATGGAC	191	+	+	-
mt7sLR	CACCACGTCAACGCAGCAAA				
it20F1	TCTGGATTGCAGTCGTCGG	209	-	+	+
it20R1	CTTGGCGATACCTCCTGAT				
it20F2	AGCCTTGGTGGTGTCTTTTG	195	-	+	+
it20R2	CAAAGAAGACGGCAGACACA				

+: Positive PCR, and -: negative PCR.

**Table 4:** Primers combinations tested for the set up of the ultra-fast duplex PCR and expected amplicons size.

Duplex PCRs	Primer Pairs	Size (bp)	Expected Specificity		
			*L. major*	*L. tropica*	*L. infantum*
A	mt22F1	366	+	+	-
	mt22R1				
	it20F1	209	-	+	+
	it20R1				
B	mt22F2	319	+	+	-
	mt22R2				
	it20F2	195	-	+	+
	it20R2				
C	mt22F1	366	+	+	-
	mt22R1				
	it20F2	195	-	+	+
	it20R2				
D	mt22F2	319	+	+	-
	mt22R2				
	it20F1	209	-	+	+
	it20R1				
E	mt22F2	333	+	+	-
	mt22R1				
	it20F1	209	-	+	+
	it20R1				
F	mt22F2	333	+	+	-
	mt22R1				
	it20F2	195	-	+	+
	it20R2				
G	mt30F	350	+	+	-
	mt30R				
	it20F1	209	-	+	+
	it20R1				
H	mt30F	350	+	+	-
	mt30R				
	it20F2	195	-	+	+
	it20R2				

+: positive PCR; -: Negative PCR.

## Data Availability

Public data used in this study are available on tritrypdb (www.tritrypdb.org, accessed on 24 October 2023).
